# Loneliness, Social Isolation and Their Difference: A Cross-Diagnostic Study in Persistent Depressive Disorder and Borderline Personality Disorder

**DOI:** 10.3389/fpsyt.2020.608476

**Published:** 2020-12-17

**Authors:** Tabea Nenov-Matt, Barbara B. Barton, Julia Dewald-Kaufmann, Stephan Goerigk, Stephanie Rek, Katharina Zentz, Richard Musil, Andrea Jobst, Frank Padberg, Matthias A. Reinhard

**Affiliations:** ^1^Department of Psychiatry and Psychotherapy, LMU University Hospital Munich, Munich, Germany; ^2^Hochschule Fresenius, University of Applied Sciences, Munich, Germany

**Keywords:** loneliness, social isolation, childhood maltreatment, rejection sensitivity, persistent depressive disorder, borderline personality disorder

## Abstract

**Background:** Interpersonal difficulties are a key feature of persistent depressive disorder (PDD) and borderline personality disorder (BPD). Caught in a vicious circle of dysfunctional interpersonal transaction, PDD and BPD patients are at great risk of experiencing prolonged loneliness. Loneliness, in turn, has been associated with the development of mental disorders and chronic illness trajectories. Besides, several factors may contribute to the experience of loneliness across the lifespan, such as social network characteristics, a history of childhood maltreatment (CM), and cognitive-affective biases such as rejection sensitivity (RS). This cross-diagnostic study approached the topic of perceived loneliness by comparing PDD and BPD patients with healthy controls (HC) in its interplay with symptom burden, social network characteristics, RS as well as CM.

**Method:** Thirty-four PDD patients (DSM-5; 15 female, M_age_ = 38.2, SD = 12.3), 36 BPD patients (DSM-5; 19 female, M_age_ = 28.8, SD = 9.2), and 70 age- and gender-matched HC were assessed cross-sectionally using the following self-report measures: UCLA Loneliness Scale, Social Network Index (SNI; size, diversity, and embeddedness), Beck Depression Inventory (BDI-II), Borderline Symptom List (BSL-23), Childhood Trauma Questionnaire (CTQ), and Rejection Sensitivity Questionnaire (RSQ).

**Results:** Both patient groups reported significantly higher levels of perceived loneliness, symptom severity, and smaller social network characteristics compared to HC. Loneliness was significantly correlated with severity of self-reported clinical symptoms in PDD and at trend level in BPD. Besides, loneliness tended to be related to social network characteristics for all groups except PDD patients. Both PDD and BPD patients showed higher RS as well as CTQ scores than HC. A history of emotional abuse and emotional neglect was associated with loneliness, and this association was mediated by RS as demonstrated by an exploratory mediation analysis.

**Discussion:** Loneliness is highly prevalent in PDD and BPD patients and contributes to the overall symptom burden. Interestingly, loneliness showed an association with prior experiences of CM as well as current RS. We therefore propose a comprehensive model on how intra- und interpersonal aspects may interplay in the dynamics of loneliness in light of CM. Finally, this model may have further implications for psychotherapeutic interventions.

## Introduction

Interpersonal difficulties are highly prevalent in several complex psychiatric disorders, e.g., persistent depressive disorder (PDD) and borderline personality disorder (BPD). These are reflected in dysfunctional social interactions, low social integration, and insufficient social support (1–3). Regarding interpersonal styles, for instance, individuals with PDD tend to have more hostile, hostile-submissive, and hostile-dominant interpersonal behaviors than normative and other clinical samples (4–6). Regarding BPD, the first two diagnostic criteria directly refer to difficulties in making and maintaining interpersonal relationships (7). Over time, these interpersonal difficulties can elicit rejection from others, ultimately leading to poor-quality relationships and social withdrawal (8). The strain of PDD and BPD patients' relationships can be assumed to increase the likelihood and severity of experiencing loneliness: caught in this vicious circle of dysfunctional interpersonal transaction, PDD and BPD patients are likely at great risk of experiencing prolonged loneliness (9, 10). Loneliness, defined as a perceived mismatch between existing social relationships and subjective social ideals (11), develops when our needs for social belongingness are not sufficiently met (12). It is different from its positive counterpart called solitude and the formal criterion of social isolation (13). As loneliness influences affective, cognitive, and behavioral processes (14), it can in turn lead to a range of interpersonal problems and result in social isolation (15). It has even been suggested that the dysfunctional interpersonal processes of lonely individuals contribute to mental health problems [e.g., (16)]. Therefore, a vicious circle can be assumed with loneliness being both a causal as well as a maintaining factor of PDD and BPD.

Different theories aim at explaining the phenomenon of loneliness. Psychodynamic models of loneliness suggest that several factors across the lifespan may contribute to the experience of loneliness with early experiences during childhood, i.e., childhood maltreatment (CM), being of major importance (17, 18). As outlined in the attachment hypothesis on loneliness (19), adult interpersonal difficulties may result from non-secure attachment representations as well as a history of early interpersonal trauma (20). In line with this, CM experiences (e.g., emotional maltreatment, physical abuse and neglect, sexual abuse) have been found to predict adult loneliness (21–23) and lonely adolescents report higher levels of parental rejection during childhood compared to non-lonely adolescents (24). Taken together, prior studies suggest that loneliness later in life may be related to early experiences of CM.

Based on cognitive-behavioral models, cognitive-affective biases such as rejection sensitivity (RS) may also contribute to the development and maintenance of loneliness (25, 26). RS is defined as a personality disposition to anxiously expect, readily perceive, and overreact to rejection (27). As loneliness threatens the need for social belongingness, it is argued to serve as an aversive, yet adaptive, signal to promote social reconnection in a regulatory loop (28). Thus, short-term loneliness activates a series of social-cognitive processes that aim to provide a behavioral response to re-establish social contact (25, 29). However, prolonged loneliness may lead to a self-preservation bias in cognitive processes (such as RS) to protect the lonely individual in socially threatening environments (30). In line with this, previous research suggests that biased social cognitions are key characteristics of prolonged loneliness (31). These social-cognitive biases are assumed to affect attention, interpretation, and memory of social stimuli to increase attention toward socially relevant information (32). They may ultimately affect behavioral processes, resulting in a self-reinforcing loop in which lonely individuals actively distance themselves and elicit behaviors from others that validate their rejection expectations (25, 33).

Based on the assumption that loneliness arises from deficits in social relationships, prior research has investigated whether perceived loneliness may be associated with social network characteristics (34). According to the cognitive discrepancy perspective on loneliness, the decisive criterion for loneliness is subjective preference or expectation, making social isolation neither a necessary nor sufficient requirement for loneliness (11). Lonely and non-lonely individuals engage in similar activities with equivalent time alone during the day (35). Neither a high number of social contacts protects one from feeling lonely (36), nor is loneliness necessarily associated with a small number of social contacts (37). However, previous findings were heterogeneous, as other studies found individuals with less frequent participation in social activities at greatest risk of being lonely (38–40). Therefore, other aspects of the social network, i.e., its composition and functioning, may be more important than the network size. Jones (41) showed that while the total amount of social contact does not vary between lonely and non-lonely individuals, the type of contact does: as non-lonely individuals engage in more interactions with friends and family, lonely people engage in fewer interactions with intimates and more interactions with strangers and acquaintances. This implies that human beings need to feel connected to significant others and that the mere physical presence of others is not sufficient (42).

Considering the interplay of loneliness, depressive symptoms, and pervasive interpersonal difficulties, as well as their similar roots in trauma history, it appears fruitful to further investigate the role of loneliness in patients with PDD and BPD. In terms of loneliness and related factors, however, PDD and BPD patients may share characteristic features but have not been directly compared to date.

As outlined above, loneliness is argued to arise when people perceive their social relationships as somehow deficient. As PDD and BPD have been linked to severe interpersonal disturbances, both patient groups are likely to perceive the quality and/or quantity of their social bonds to be unsatisfactory. Affective, cognitive, and interpersonal characteristics of PDD and BPD patients may hinder social reconnection and thus maintain loneliness, as a diminished capacity for pro-social behavior and interpersonal understanding is often related to increased feelings of loneliness (43). Enduring feelings of loneliness can thus be assumed highly prevalent in PDD and BPD patients, negatively impacting illness severity and course.

More specific findings regarding loneliness have been observed in BPD patients. Besides increased levels of loneliness, BPD patients have smaller social networks compared to HC (9, 44). Furthermore, the networks of BPD patients include a great number of former romantic partners (45). As BPD patients show a comparable trauma load, chronicity, and treatment resistance as PDD patients, comparing these two patient groups is especially valuable. Furthermore, depression is highly prevalent in BPD patients (46).

In summary, this study aimed to contribute to a better understanding of loneliness and its association with symptom burden, social network characteristics, potential cognitive-affective biases (e.g., RS), and CM in PDD patients in comparison with BPD patients and HC. Clarifying the psychological and interpersonal correlates of PDD and BPD as well as their relative influence on the development and maintenance of the disorder is particularly important given the limited effectiveness of current treatments. A deeper understanding of loneliness in PDD and BPD may guide clinical decision making and intervention efforts.

## Materials and Methods

### Participants

Data were derived from 140 individuals who participated in a study assessing the response to social exclusion and rejection at the Department of Psychiatry and Psychotherapy of the LMU University Hospital, Munich. The study followed the Declaration of Helsinki and was approved by the Research Ethics Board of the Ludwig Maximilians University, Faculty of Medicine, Munich (#281-11). Participants provided written informed consent prior to study participation.

Both PDD patients and BPD patients were recruited at the Department of Psychiatry and Psychotherapy of the LMU University Hospital, Munich and by advertisements. Patients were included if they fulfilled the diagnoses PDD or BPD following DSM-5 criteria (7). General exclusion criteria included acute suicidality, mania, psychosis, substance use disorders as a primary diagnosis, taking sedative medication regularly, pregnancy, or current breastfeeding. Comorbid psychiatric disorders were assessed according to DSM-IV by experienced clinical psychologists who were trained in conducting interviews using the German version of the Structured Clinical Interview for DSM-IV [SCID-I, (47, 48); SCID-II, (49, 50)].

Two groups of HC were recruited by advertisements to age and gender-match both patient groups (HC_PDD_ and HC_BPD_). Besides the mentioned general exclusion criteria, additional exclusion criteria for HC were any current or lifetime psychiatric diagnosis, BDI-II > 11, psychiatric medication, or psychotherapy within the past 10 years.

### Loneliness

Loneliness was assessed using the German adaption of the UCLA Loneliness Scale (UCLA-LS) based on a revised version of the original UCLA-LS (51, 52). It consists of 20 items examining the frequency and intensity of loneliness-related experiences, both positively worded (e.g., “There are people I feel close to.”), as well as negatively worded (e.g., “People are around me but not with me.”). Responses range from 1 (not at all) to 5 (totally). A total score is formed by reversing items where needed and adding responses. The total score is divided by the number of valid items, with a mean score ranging from 1 to 5. Higher scores indicate more intense feelings of loneliness. The internal consistency in our sample was high (Cronbach's alpha: PDD:0.91; BPD:0.93; HC_PDD_:0.90; HC_BPD_:0.91).

### Social Network Characteristics

Social network characteristics were assessed using the German version of the Social Network Index [SNI, (53)]. The SNI is a self-administered instrument with 12 items assessing 12 different types of social relationships (e.g., spouse, parents, children, friends, workmates). For each type of relationship, respondents are asked how many people he/she knows and talks to at least once every 2 weeks. These questions are answered with a number between 0 and 6 or “7 or more,” except for parents, who are naturally restricted to two, and for the items on romantic partnership, where only a yes or no answer is permitted. The SNI quantifies (a) the size of the social network, (b) network diversity, and (c) the number of embedded networks. The size of the social network is defined as the total number of people with whom the respondent has regular contact (i.e., speaks at least once every 2 weeks). Social network diversity quantifies the number of social roles, i.e., the number of social relationship domains in which the respondent has regular contact with at least one person. The number of embedded networks is a measurement reflecting the number of different network domains within which the respondent has at least four high-contact people. The family roles are collapsed into a single domain for this measure. High scores indicate large size, diversity, or a high number of embedded networks.

### Severity of Depressive and Borderline Symptoms

Severity of depressive symptoms was evaluated using the German version of the Beck Depression Inventory, revised version [BDI-II, (54, 55)] as a 21-item self-report measure. The total score ranges from 0 to 63 with higher scores indicating greater severity. The BDI-II has a high internal consistency (Cronbach's alpha > 0.84) and a good test-retest reliability (*r* > 0.75) (56).

The Montgomery–Åsberg Depression Rating Scale [MADRS, (57)] is an observer-based interview that assesses the severity of 10 depressive symptoms with a total score between 0 and 60. Internal consistency is high (Cronbach's alpha = 0.85) (58).

BPD severity was measured using the short version of the Borderline Symptom List [BSL-23, (59)]. The BSL-23 assesses self-reported severity of borderline-specific symptomatology during the past week. It contains 23 items rated on a 5-point Likert scale that are summarized and divided by the number of items to form a total score from 0 to 92. The BSL-23 has a high internal consistency (Cronbach's alpha = 0.94–0.97), high test-retest reliability (*r* = 0.82) and is very reliable in the diagnosis of BPD (60).

### Rejection Sensitivity

RS was measured with the German version of the Rejection Sensitivity Questionnaire for adults [RSQ, (61)]. Respondents are presented with 20 scenarios in which they have to make a request of a significant other (e.g., parent, friend, romantic partner). They are then asked to rate both their anxiety and their expectation to be rejected in the particular scenario on a 6-point Likert scale. Scores for each scenario are multiplied and then divided by the number of scenarios. Total scores range from 1 to 36, with higher scores indicating greater RS. The RSQ has a high internal consistency (Cronbach's alpha = 0.88) and a high test-retest reliability (*r* = 0.90) (61).

### Childhood Maltreatment

CM was assessed using the German version of the Childhood Trauma Questionnaire, short-form [CTQ, (62–64)]. The CTQ is a 28-item self-report measure consisting of statements about experiences of sexual, physical, and emotional abuse as well as physical and emotional neglect during childhood and adolescence. Respondents are asked to indicate to which extent these statements describe their experiences, rating items on a 5-point Likert scale from 1 (never true) to 5 (very often true). Item scores are added to several subscales ranging from 5 to 25, with higher scores indicating more frequent childhood abuse and/or neglect. For the German version of the CTQ the internal consistency of all scales (apart from physical neglect) is high (Cronbach's alpha > 0.80). The psychometric properties of the German version are similar to the American original, making it a reliable and valid screen for the retrospective assessment of CM (65).

### Data Analysis

Statistical analyses were conducted with SPSS version 25 (https://www.ibm.com/de-de/products/spss-statistics). One-way ANOVAs with four planned contrasts were applied to analyze group differences for the different measures: (1) PDD patients vs. matched HC_PDD_, (2) BPD patients vs. matched HC_BPD_, (3) PDD patients vs. BPD patients, (4) HC_PDD_ vs. HC_BPD_. As age and sex were not correlated with loneliness, these variables were not included as covariates. In the next step, correlations of loneliness with different variables were calculated within each subgroup using parametric and non-parametric methods (Pearson, Spearman) as appropriate. Due to the high number of correlations, *p*-values were adjusted according to Benjamini and Hochberg (66) for all calculated correlations. As loneliness was found to be associated with emotional abuse, emotional neglect, and RS in patients as well as in HC though in varying strength, two exploratory mediation analyses were conducted using a robust bootstrapping approach (10.000 bootstraps, PROCESS macro version 3.5) with loneliness as dependent variable, emotional abuse or emotional neglect as independent variable, and RS as mediating variable. Analyses were restricted to either the patient or to the HC subgroup due to the observed group differences in these variables.

## Results

### Sample

Thirty-four PDD patients (DSM-5; 15 female, M_age_ = 38.2, SD = 12.3), 36 BPD patients (DSM-5; 19 female, M_age_ = 28.8, SD = 9.2) and two groups of age- and gender-matched HC (in total 70 HC) were assessed cross-sectionally. Groups differed significantly regarding age [F_(3, 136)_ = 8.6, *p* < 0.001]: PDD patients were significantly older than BPD patients (*p* = 0.002) as were HC_PDD_ compared to HC_BPD_, respectively. Furthermore, groups differed regarding years of education [F_(3, 135)_ = 7.9, *p* < 0.001], i.e., BPD patients had significantly less years of education than their matched HC (*p* = 0.02), than PDD patients (*p* = 0.004) and than HC_PDD_ (*p* < 0.001).

Patients showed a variety of comorbid disorders: 47.2% of BPD patients met criteria for a current major depressive episode with 38.9% meeting criteria for comorbid PDD. Further, 41.7% of BPD patients had a comorbid PTSD, 36.1% a comorbid social anxiety disorder, and 19.4% of BPD patients an eating disorder. 47.1% of PDD patients met criteria for current major depressive episode, 17.6% for social anxiety disorder, and 14.7% for PTSD.

### Loneliness and Social Network Characteristics

Both PDD and BPD patients reported significantly higher levels of perceived loneliness than the matched HC group (see Tables 1, 2). BPD patients reported even more feelings of loneliness than PDD patients. Besides, HC groups differed regarding loneliness, with higher loneliness scores in HC_PDD_ compared to HC_BPD_. Social network characteristics (i.e., size, diversity, and number of embedded networks) differed between both patient groups and the matched HC groups, but neither between PDD and BPD patients nor between HC groups.

**Table 1:** Loneliness, social network characteristics, clinical symptoms, and childhood maltreatment: mean scores and standard deviation together with results of univariate ANOVA.

**Measure**	**PDD**	**BPD**	**HC_**PDD**_**	**HC_**BPD**_**	**ANOVA**	
					**Global F**	***p***
UCLA-Loneliness	2.7 (0.7)	3.0 (0.7)	1.7 (0.5)	1.4 (0.4)	57.7	< 0.001^***^
SNI-Size	8.8 (5.3)	9.6 (7.0)	20.4 (8.4)	21.9 (10.8)	25.3	< 0.001^***^
SNI-Diversity	3.7 (1.6)	3.4 (1.6)	5.9 (1.9)	5.6 (1.8)	19.2	< 0.001^***^
SNI-Embeddedness	0.8 (0.8)	1.0 (1.1)	2.4 (1.2)	2.4 (1.5)	18.0	< 0.001^***^
BDI-II	25.5 (11.3)	31.4 (10.7)	1.8 (2.7)	2.4 (2.9)	128.8	< 0.001^***^
MADRS	18.0 (7.5)	15.9 (7.0)	0.6 (1.2)	0.5 (1.0)	96.1	< 0.001^***^
BSL-23	1.0 (0.7)	2.0 (0.9)	0.2 (0.2)	0.2 (0.2)	85.8	< 0.001^***^
RSQ	12.8 (4.1)	16.6 (5.7)	6.3 (2.9)	5.9 (3.0)	57.3	< 0.001^***^
CTQ-Emotional abuse	13.3 (5.5)	14.7 (4.9)	7.0 (3.5)	6.8 (2.4)	33.5	< 0.001^***^
CTQ-Physical abuse	6.7 (2.5)	8.9 (5.3)	5.7 (2.0)	5.4 (1.1)	8.6	< 0.001^***^
CTQ-Sexual abuse	6.6 (4.2)	8.0 (5.2)	5.6 (1.6)	5.2 (0.7)	4.8	0.003^**^
CTQ-Emotional neglect	15.4 (5.0)	16.5 (4.9)	8.3 (3.1)	7.7 (3.0)	45.0	< 0.001^***^
CTQ-Physical neglect	7.9 (2.5)	10.1 (3.7)	6.5 (2.6)	6.4 (1.6)	14.8	< 0.001^***^

*PDD, persistent depressive disorder; BPD, borderline personality disorder; HC, healthy controls; SNI, social network index; BDI-II, Beck depression inventory; MADRS, Montgomery-Åsberg Depression Rating Scale; BSL-23, borderline symptom list; RSQ, rejection sensitivity questionnaire; CTQ, childhood trauma questionnaire*;

** *p < 0.01*,

*** *p < 0.001*.

**Table 2:** Loneliness, social network characteristics, clinical symptoms, and childhood maltreatment: results and effect size (Cohen's *d*) of planned contrasts between patient groups and their matched healthy controls.

**Measure**	**Contrast PDD vs. HC_PDD_**			**Contrast BPD vs. HC_BPD_**			**Contrast PDD vs. BPD**			**Contrast HC_PDD_ vs. HC_BPD_**		
	***t***	***p***	***d***	***t***	***p***	***d***	***t***	***p***	***d***	***t***	***p***	***d***
UCLA-Loneliness	6.8	< 0.001^***^	1.8	11.2	< 0.001^***^	2.8	2.1	0.04^*^	−0.4	2.1	0.04^*^	0.7
SNI-Size	5.8	< 0.001^***^	−1.7	6.4	< 0.001^***^	−1.4	0.4	0.68	−0.1	0.8	0.43	−0.2
SNI-Diversity	5.2	< 0.001^***^	−1.3	5.4	< 0.001^***^	−1.3	0.9	0.45	0.2	0.7	0.47	0.2
SNI-Embeddedness	5.3	< 0.001^***^	−1.5	5.0	< 0.001^***^	−0.9	0.6	0.53	−0.2	0.1	0.90	−0.0
BDI-II	12.2	< 0.001^***^	2.9	15.2	< 0.001^***^	3.7	3.0	0.003^**^	−0.5	0.3	0.75	−0.2
MADRS	12.2	< 0.001^***^	3.2	11.6	< 0.001^***^	3.1	1.6	0.11	0.3	0.1	0.93	0.1
BSL-23	6.3	< 0.001^***^	1.6	13.8	< 0.001^***^	2.8	7.3	< 0.001^***^	−1.2	0.1	0.96	−0.0
RSQ	6.6	< 0.001^***^	1.8	11.1	< 0.001^***^	2.3	3.8	< 0.001^***^	−0.8	0.4	0.71	0.1
CTQ-Emotional abuse	6.1	< 0.001^***^	1.4	7.9	< 0.001^***^	2.0	1.4	0.16	−0.3	0.2	0.80	0.1
CTQ-Physical abuse	1.3	0.20	0.4	4.6	< 0.001^***^	0.9	2.9	0.005^**^	−0.5	0.4	0.68	0.2
CTQ-Sexual abuse	1.2	0.22	0.3	3.5	0.001^**^	0.8	1.7	0.09	−0.3	0.5	0.64	0.3
CTQ-Emotional neglect	7.2	< 0.001^***^	1.7	9.14	< 0.001^***^	2.2	1.1	0.25	−0.2	0.6	0.56	0.2
CTQ-Physical neglect	2.1	0.03^*^	0.5	5.9	< 0.001^***^	1.3	3.4	0.001^**^	−0.7	0.1	0.88	0.0

*PDD, persistent depressive disorder; BPD, borderline personality disorder; HC, healthy controls; SNI, social network index; BDI-II, Beck depression inventory; MADRS, Montgomery-Åsberg Depression Rating Scale; BSL-23, borderline symptom list; RSQ, rejection sensitivity questionnaire; CTQ, childhood trauma questionnaire*;

* *p < 0.05*,

** *p < 0.01*,

*** *p < 0.001*.

### Severity of Depressive and Borderline Symptoms

Depressive symptoms were more prevalent in both patient groups than in their matched HC, and BPD patients had higher BDI-II scores than PDD patients but did not differ in the observer-rated measure (MADRS, see Tables 1, 2). Similarly, both patient groups reported higher borderline symptom scores than their matched HC (BSL-23), with a significant difference between PDD and BPD patients, i.e., moderate scores in PDD and high scores in BPD (67).

### Rejection Sensitivity and Childhood Maltreatment

Both patient groups showed significantly higher RS scores than their HC group, and BPD patients had significantly higher RS scores than PDD patients. Regarding CM, PDD patients reported more often emotional abuse, emotional neglect, and physical neglect than their matched HC. In contrast, BPD patients reported a higher CM load on all CTQ subscales than their matched HC. BPD patients showed higher levels of physical abuse and physical neglect compared to PDD patients (see Tables 1, 2).

### Associations Between Loneliness, Social Network, Clinical Symptoms, and Childhood Maltreatment

Loneliness and social network features correlated significantly negatively within HC_PDD_ and at trend level within HC_BPD_ after FDR correction (size: HC_PDD_: *r* = −0.42, p_FDR_ = 0.04; HC_BPD_: *r* = −0.35, p_FDR_ = 0.07; diversity: HC_PDD_: *r* = −0.43, p_FDR_ = 0.04; embeddedness: HC_PDD_: *r* = −0.42, p_FDR_ = 0.04; HC_BPD_: *r* = −0.40, p_FDR_ = 0.05; see Table 3). Furthermore, loneliness showed an inverse correlation at trend level within the BPD group with social network size (*r* = −0.34, p_FDR_ = 0.08) and diversity (*r* = −0.37, p_FDR_ = 0.06). In contrast, PDD patients showed no inter-correlation of social network features and loneliness at all. Loneliness and severity of self-reported depressive symptoms correlated significantly in PDD patients (*r* = 0.55, p_FDR_ = 0.008) and at trend level in BPD patients (*r* = 0.38, p_FDR_ = 0.06) and their matched HC_BPD_ (*r* = 0.34, p_FDR_ = 0.08). Loneliness was significantly correlated with the BSL-23 scores (after removing the BSL-23 loneliness item) in the PDD sample (*r* = 0.44, p_FDR_ = 0.04) and at trend level in the other groups (BPD: *r* = 0.32, p_FDR_ = 0.09; HC_PDD_: *r* = 0.38, p_FDR_ = 0.06; HC_BPD_: *r* = 0.37, p_FDR_ = 0.06). Additionally, loneliness showed a significant positive correlation with RS in both patient groups and HC (PDD: *r* = 0.54, p_FDR_ = 0.008; BPD: r = 0.42, p_FDR_ = 0.04; HC_PDD_: *r* = 0.74, p_FDR_ < 0.001; HC_BPD_: *r* = 0.54, p_FDR_ = 0.008). Regarding loneliness and CM, only the correlation with emotional abuse reached significance in the PDD sample (*r* = 0.44, p_FDR_ = 0.04), whereas correlations with emotional abuse and emotional neglect were significant in BPD patients (emotional abuse: *r* = 0.46, p_FDR_ = 0.02; emotional neglect: *r* = 0.53, p_FDR_ = 0.008). In the HC group, loneliness was significantly correlated with emotional neglect in both HC groups (HC_PDD_: *r* = 0.61, p_FDR_ = 0.003; HC_BPD_: *r* = 0.49, p_FDR_ = 0.02) and with physical neglect in HC_PDD_ (*r* = 0.52, p_FDR_ = 0.01).

**Table 3:** Correlation coefficients of loneliness with social network characteristics, clinical symptoms, and childhood maltreatment.

	**PDD**			**BPD**			**HC_**PDD**_**			**HC_**BPD**_**		
**UCLA-Loneliness**	***r***	***p***	***p_***FDR***_***	***r***	***p***	***p_***FDR***_***	***r***	***p***	***p_***FDR***_***	***r***	***p***	***p_***FDR***_***
SNI-Size	−0.15	0.41	0.46	−0.34	0.04	0.08	-0.42	0.01	0.04^*^	−0.35	0.04	0.07
SNI-Diversity	−0.19	0.28	0.34	−0.37	0.02	0.06	-0.43	0.01	0.04^*^	−0.24	0.16	0.21
SNI-Embeddedness	−0.07	0.69	0.73	−0.25	0.14	0.20	-0.42	0.01	0.04^*^	−0.40	0.02	0.05
BDI-II	0.55	0.001	0.008^**^	0.38	0.02	0.06	0.08	0.67	0.73	0.34	0.04	0.08
MADRS	0.41	0.02	0.05	0.22	0.19	0.23	-0.08	0.71	0.73	0.31	0.08	0.12
BSL-23	0.44	0.009	0.04^*^	0.32	0.06	0.09	0.38	0.03	0.06	0.37	0.03	0.06
RSQ	0.54	0.001	0.008^**^	0.42	0.01	0.04^*^	0.74	< 0.001	< 0.001^***^	0.54	0.001	0.008^**^
CTQ-Emotional abuse	0.44	0.009	0.04^*^	0.46	0.004	0.02^*^	0.30	0.08	0.12	0.30	0.08	0.12
CTQ-Physical abuse	0.17	0.35	0.40	0.35	0.04	0.08	0.19	0.27	0.06	0.16	0.34	0.40
CTQ-Sexual abuse	0.00	0.99	0.99	0.34	0.04	0.08	0.32	0.07	0.11	0.24	0.17	0.21
CTQ-Emotional neglect	0.25	0.16	0.21	0.53	0.001	0.008^**^	0.61	< 0.001	0.003^**^	0.49	0.003	0.02^*^
CTQ-Physical neglect	0.23	0.19	0.23	0.38	0.02	0.06	0.52	0.002	0.01^*^	−0.07	0.70	0.73

*PDD, persistent depressive disorder; BPD, borderline personality disorder; SNI, social network index; BDI-II, Beck depression inventory; MADRS, Montgomery-Åsberg Depression Rating Scale; BSL-23, borderline symptom list; RSQ, rejection sensitivity questionnaire; CTQ, childhood trauma questionnaire*;

* *p < 0.05*,

** *p < 0.01*,

*** *p < 0.001 before and after false discovery rate (FDR) correction according to Benjamini Hochberg*.

When comparing the strengths of the correlation coefficients between groups, analyses revealed that BDI-II and MADRS showed a significantly stronger correlation with loneliness in PDD patients compared to HC_PDD_ (BDI-II: *Z* = 2.11, *p* = 0.03; MADRS: *Z* = 2.03, *p* = 0.04). Furthermore, there was a trend that emotional neglect correlated stronger with loneliness in HC_PDD_ compared to PDD patients (*Z* = 1.79, *p* = 0.07). Finally, physical neglect was significantly less associated with loneliness in HC_BPD_ compared to HC_PDD_ (*Z* = 2.58, *p* = 0.01) and to BPD patients (*Z* = 1.91, *p* = 0.06). No other significant differences between correlation coefficients were detected.

### Mediation Analyses

**Figure 1:**
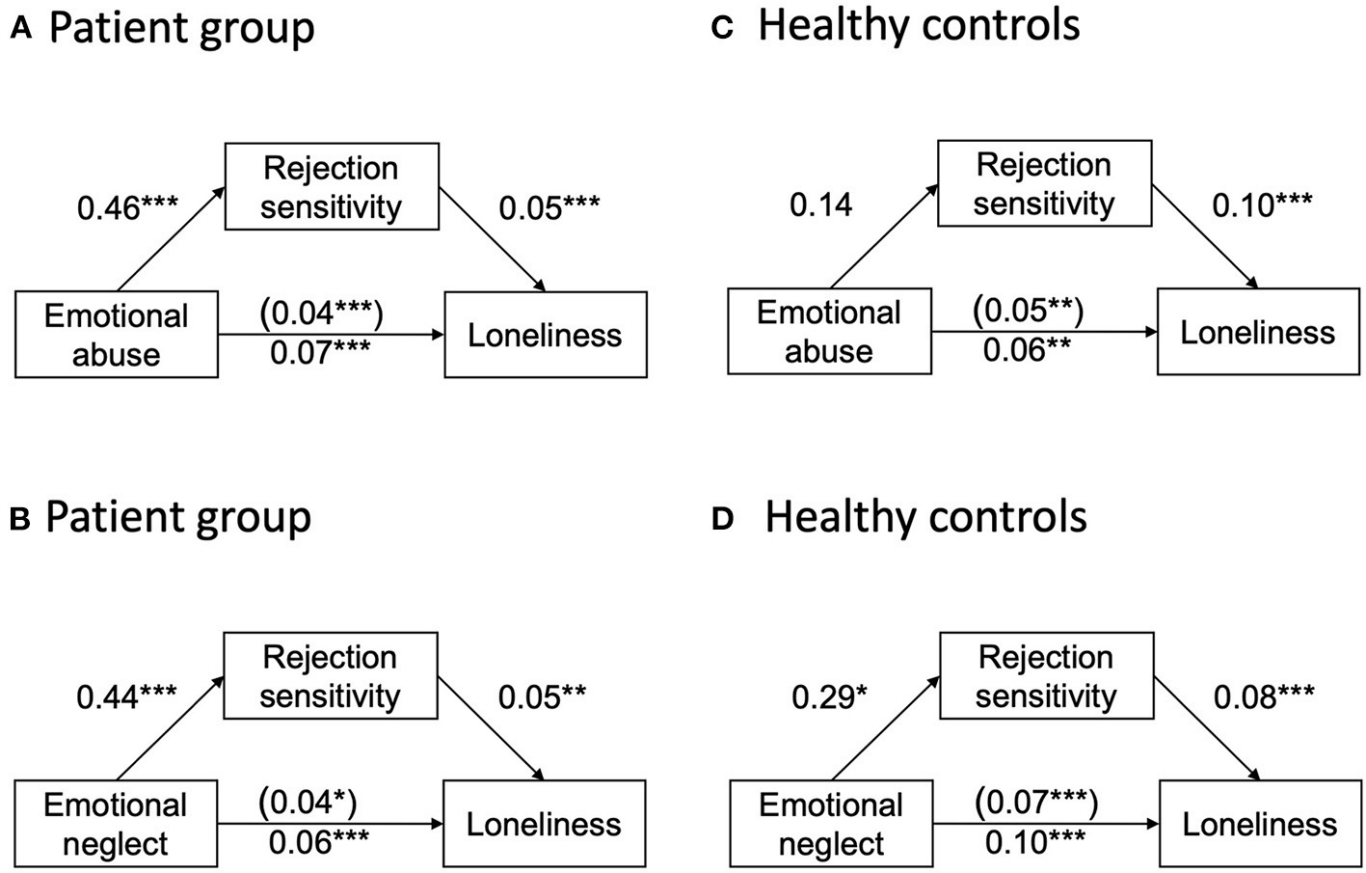
**(A)** and **(C)**: Unstandardized regression coefficients for the relationship between emotional abuse and loneliness as mediated by rejection sensitivity for the patient **(A)** and healthy control sample **(C)**. The regression coefficient between emotional abuse and loneliness, controlling for rejection sensitivity, is in parentheses; **(B)** and **(D)**: Unstandardized regression coefficients for the relationship between emotional neglect and loneliness as mediated by rejection sensitivity for the patient **(B)** and healthy control sample **(D)**. The regression coefficient between emotional neglect and loneliness, controlling for rejection sensitivity, is in parentheses, *p < 0.05, **p < 0.01, ***p < 0.001.

In the patient sample, we found that the total effect of emotional abuse on loneliness when not including RS was positive and significant (*b* = 0.07, SE = 0.02, *p* < 0.001). Second, the path from emotional abuse to RS was positive and statistically significant (*b* = 0.46, SE = 0.11, *p* < 0.001). Third, when predicting loneliness from emotional abuse and RS, the effect of RS on loneliness was positive and significant (*b* = 0.05, SE = 0.02, *p* < 0.001) as was the path from emotional abuse to loneliness (*b* = 0.04, SE = 0.02, *p* < 0.001). Finally, the indirect effect of emotional abuse on loneliness was found to be statistically significant [indirect effect *b* = 0.02, 95% C.I. (0.01, 0.04)], indicating a significant mediation effect of RS (see Figure 1A).

When using emotional neglect as independent variable, the total effect of emotional neglect on loneliness (when not including RS) was positive and significant (*b* = 0.06, SE = 0.02, *p* < 0.001). Second, the path from emotional neglect to RS was positive and statistically significant (*b* = 0.44, SE = 0.12, *p* < 0.001). Third, when predicting loneliness from emotional neglect and RS, the effect of RS on loneliness was positive and significant (b = 0.05, SE =.02, p =.001) as was the path from emotional neglect to loneliness (*b* = 0.04, SE = 0.02, *p* = 0.03). Finally, the indirect effect of emotional neglect on loneliness was found to be statistically significant [indirect effect *b* = 0.02, 95% C.I. (0.01, 0.05)], indicating a significant mediation effect of RS (see Figure 1B).

In contrast, when repeating the analyses for the HC group, no significant mediation effect of RS could be found for the association of emotional abuse with loneliness [indirect effect: *b* = 0.01, 95% C.I. (−0.02, 0.05), see Figure 1C]. With emotional neglect as independent variable, however, the total effect of emotional neglect on loneliness (when not including RS) was positive and significant (*b* = 0.10, SE = 0.02, *p* < 0.001). Second, the path from emotional neglect to RS was positive and statistically significant (*b* = 0.29, SE = 0.11, *p* = 0.01). Third, when predicting loneliness from emotional neglect and RS, the effect of RS on loneliness was positive and significant (*b* = 0.08, SE = 0.01, *p* < 0.001) as was the path from emotional neglect to loneliness (*b* = 0.07, SE = 0.01, *p* < 0.001). Finally, the indirect effect of emotional neglect on loneliness was found to be statistically significant [indirect effect *b* = 0.02, 95% C.I. (0.01, 0.05)] indicating a significant mediation effect of RS (see Figure 1D).

## Discussion

To our knowledge, the present study investigated loneliness and its underpinnings in terms of symptom burden, social network characteristics, RS, and patients' history (i.e., CM) in a cross-diagnostic approach comparing PDD and BPD patients and HC for the first time. We aimed at understanding the impact of the common phenomenon of loneliness on the development and maintenance of PDD and BPD to derive possible implications for intervention efforts.

Loneliness is of high societal interest and appears to be a major risk factor in mental health (68). Our findings confirmed that both PDD and BPD patients report higher levels of loneliness than HC. Besides, PDD and BPD patients reported significantly more depressive symptoms and borderline symptoms than their respective matched HC group. BPD patients reported even higher depression and borderline scores than PDD patients, consistent with prior research showing that BPD patients rate their depressive symptoms higher (69). High levels of loneliness were associated with greater symptom severity of depression and BPD in both patient groups, again confirming previous findings (44, 70). This indicates that the subjective perception and evaluation of social relationships might play an important role in the development and maintenance of mental disorders. While loneliness is known as a specific risk factor for depression (71, 72), loneliness and depression are discussed as two distinct phenomena that are associated with each other (73). Evidence holds that loneliness might impact illness trajectory and treatment outcome in depression (74). Further, loneliness has been discussed as a core experience of BPD patients (44) as it is closely linked to the feeling of inner emptiness which is a diagnostic criterion in BPD [i.e., diagnostic criterion 7; (7)]. As expected, SNI scores were significantly lower in both patient groups when compared to HC. To date, knowledge about social networks in PDD and BPD is still limited; however, our results are consistent with previous research regarding patients with PDD (75) and BPD (9). Social isolation has been discussed as a risk for depression (76, 77), e.g., people with PDD appear to have smaller social networks than the general population and patients with other mental disorders (75). Similarly, BPD patients are found to have smaller networks (9) and less satisfactory social integration (78) compared to HC.

In our study, loneliness and social network size were negatively correlated in BPD patients and both HC groups (at least at trend level after FDR correction), but not in PDD patients. One possible explanation could be that PDD is considered to be maintained by a longstanding and pervasive pattern of interpersonal avoidance, fueled by interpersonal fears such as RS. PDD patients are considered to have a “wall” around them that hinders them to perceive their interactions with others (8). Hence, the perceived loneliness of PDD patients may not depend on objective social indices, as PDD patients are perceptually disconnected from others. Furthermore, although interacting with others might end loneliness on the one hand, the potential risk of rejection might promote anxiety and hyperarousal on the other, which might be considered even worse than loneliness (79). Consistent with this, prior research has found that social interactions (16) and even the simple exposure to pleasant depictions of people (80) are more rewarding for individuals low than high in loneliness. After feeling lonely, social company was judged more negatively, predicting the frequency with which company was avoided (72). This suggests that the negative appraisal of social relationships and subsequent social withdrawal may play a role in the development of psychopathology. The dynamics between feeling lonely, being socially isolated, and negatively appraising social company may therefore represent a self-reinforcing loop. Whereas a bigger social network may be helpful in BPD patients and HC to protect from loneliness, this may not be the case in PDD. The self-protective behavior of social withdrawal prompted by fearful sensations may rather produce a self-fulfilling prophecy in which actual rejection is elicited from others (81, 82), moving lonely individuals further toward the periphery of their social networks (83, 84). Simply increasing social contact, networks, or social roles in PDD may therefore not be sufficient to mitigate loneliness. Consistent with previous research, BPD patients' loneliness correlated negatively at trend level with social network size and diversity (after FDR correction) (9). BPD patients are considered to be more ambivalent and may switch between social withdrawal and clinging behavior (61). As BPD patients may not show the perceptual disconnection from others compared to PDD patients, regular contact with a high number of people seems relevant in regard to loneliness.

Another individual factor closely related to both previous experiences in relationships as well as personality features is RS. As expected, both patient groups showed higher RS scores than HC. These results are in line with previous research that found both BPD (61, 78, 85) and PDD patients (86) to experience increased RS. Further, loneliness was correlated with RS in both patient groups and HC suggesting that RS may be an unspecific factor for the experience of loneliness. These findings are in line with previous research linking loneliness to higher self-reported anticipation of rejection (33, 86).

Finally, we analyzed the interaction between loneliness and CTQ subscales to investigate a potential origin of loneliness in CM. In line with previous research, both patient groups reported higher CTQ scores compared to their matched HC group (87). PDD patients reported more often emotional neglect and emotional abuse than their matched HC as previously reported (88–90), whereas patients with BPD reported a higher trauma load on all CTQ subscales compared to their matched HC (87). Our results are in line with previous research showing that CM has far-reaching effects on adult physical and mental health (91, 92). After correcting for multiple comparisons, loneliness correlated with emotional abuse in PDD, and with both emotional abuse and emotional neglect in BPD. To date, little is known about the association of loneliness with CM in patients with PDD and BPD. Etiological models of PDD propose experiences of abuse and neglect during childhood as possible causal factors for interpersonal problems, which may contribute to aversive feelings of loneliness (93). For BPD, Gunderson (94) suggests that loneliness might develop as a consequence of abusive primary caretakers. Consistently, loneliness was found to mediate the association between CM and adult mental disorders (22). Our findings suggest that feelings of loneliness may be related to a history of CM, i.e., particularly emotional abuse and neglect, in both PDD and BPD patients. Furthermore, we observed loneliness to be associated with RS in both patient groups and HC. Thus, we further explored the interactions of these factors in mediation analyses for emotional abuse and emotional neglect which suggested a mediating role of RS in the association of loneliness and emotional abuse/neglect in the patient group and of emotional neglect in HC. However, the divergent findings between groups have to be interpreted with caution due to the decreased prevalence of CM in HC.

**Figure 2:**
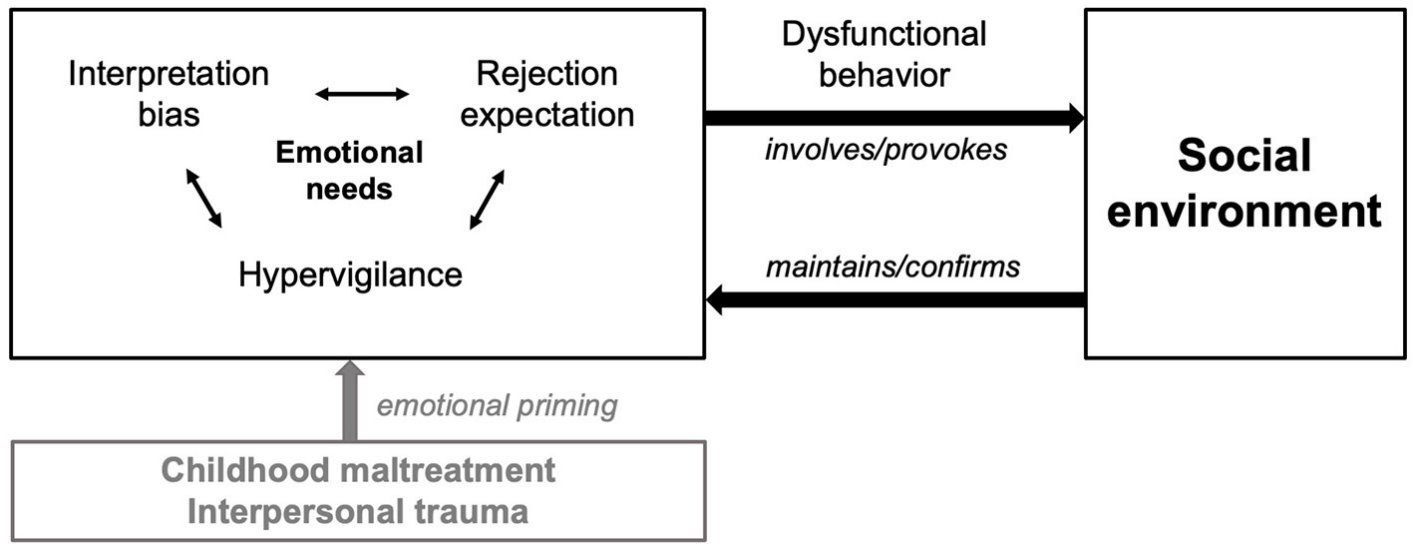
Proposed model of loneliness in PDD and BPD. Loneliness is conceptualized as an unmet emotional need rooted in a history of childhood maltreatment, e.g., emotional abuse and neglect. The interplay of intraindividual cognitive-affective biases (esp. high rejection sensitivity, comprising hypervigilance to and expectation of rejection as well as interpretation biases) contributes to dysfunctional interaction patterns with the social environment maintaining a self-reinforcing loop.

Combining our findings with previously reported models of loneliness [(25); current updates by (13, 29)], we propose an expanded hypothetical model of loneliness (see Figure 2). Loneliness is conceptualized as an unmet emotional need that arises from a history of CM (i.e., particularly emotional abuse and neglect) with cognitive-attentional, affective-feeling, sensory-perceptual, and motor-expressive aspects. Following the idea of a basic emotional need, the function of loneliness can be conceptualized in terms of evolution theory: As a social species, humans depend on a safe social surround to survive and therefore have an “innate need to belong” (12). Thus, the feeling of loneliness may serve as an alert when social connections are threatened (30). It motivates people to re-establish and maintain social contacts to increase the likelihood of survival and reproduction (30).

Horowitz et al. (95) suggested a “prototype,” including affective, cognitive, and behavioral features to conceptualize the experience of loneliness. When the individual need for social belonging—determined by the subjective level of vulnerability to social disconnection—is not met, people experience emotional distress. This distress may be triggered by external events like the loss of a significant other or by internal thoughts (e.g., “I do not belong”, “I am excluded”). Consistently, empirical research showed that lonely individuals experience predominantly negative affect (15). Weiss (19) described loneliness as a strong sense of social pain, emptiness, isolation, sadness for lack of confidants, unimportance, and worthlessness. Feeling unsafe or threatened in a social world sets off implicit hypervigilance for (additional) social threat and alters cognitive processes (25). Hypervigilance for social cues when feeling lonely could be functional in terms of choosing the most appropriate way to socially reconnect (26), as the heightened sensitivity to social verbal and non-verbal information enables the individual to react faster to perceived threats for further social isolation (25). In case maladaptive social-cognitive biases, e.g., RS, step in, and reconnection is not supported or even hampered by the environment, this regulatory loop may become a vicious circle, resulting in frequently recurrent or persistent feelings of loneliness which may maintain the course of PDD or BPD (16). Previous research suggests that biased social cognitions are one of the most pronounced characteristics of loneliness (31). Predominantly, surveillance of social environment appears to be enhanced, with lonely individuals sensing socially threatening stimuli earlier than their non-lonely peers (96). The evidence for deficits in social cognition of PDD patients is scarce (6). Regarding BPD, previous research suggests that alterations may not only be caused by a hypersensitivity to negative social information, but also a hyposensitivity to positive social stimuli, combined with reduced confidence to judge particularly positive emotional states. Interestingly, reduced confidence was related to stronger feelings of loneliness and the expectation of social rejection (97). In line with this, loneliness has been linked to higher self- and peer-reported anticipation of rejection (33, 86). The relation between loneliness and RS appears hereby to be bidirectional, with RS representing both a risk factor and a consequence of loneliness (98). This loop may even reduce prosocial behavior (43, 99), as individuals high in RS are found to engage in more dysfunctional relationship behaviors (100). Ultimately, lonely individuals may engage in a self-fulfilling expectation regarding social rejection by others which validates their negative social expectations (82) and distance themselves further (33), as they believe that the cause of social distance is beyond their control (16). Prolonged social withdrawal in child- and adulthood may limit opportunities for social reconnection (101) and impede acquisition learning of skills when relationships rupture and repair is required (15, 102).

This model could have wider clinical implications, as loneliness may represent a cross-diagnostic risk factor in mental health. Accordingly, loneliness has been identified as a target for therapeutic interventions (103) which either address (1) social or (2) cognitive factors (104). The majority of loneliness interventions focus on social factors, e.g., improving social skills, increasing the social network, or enhancing interaction quality (105, 106). Consistently, facilitating meaningful social interaction has been reported to effectively prevent and reduce depressive symptoms and relapse rates (107, 108). Social interventions are therefore a promising research avenue for alleviating loneliness in PDD and BPD patients. However, loneliness and social network characteristics are often weakly associated as observed here and by others (109). Thus, merely enhancing the frequency of social contact does not necessarily alleviate loneliness and such interventions may miss the point that loneliness has rather to do with the perception of ourselves and the quality of social interactions than with social network sizes (109). Indeed, a very recent study suggests cognitive reappraisal interventions addressing time spent alone as an effective method to alleviate loneliness (104). Thus, psychotherapeutic approaches for reducing loneliness should focus on dysfunctional interpersonal processes and maladaptive social cognitions, stemming from early interpersonal trauma (CM). One example of such a therapeutic approach is the Cognitive Behavioral Analysis System of Psychotherapy (CBASP) that has been specifically designed for the treatment of PDD. In brief, CBASP encompasses techniques like the “situational analysis” that focuses on actual automatic thoughts, cognitive biases, and behavioral patterns, and largely aims at improving the quality of interpersonal situations (8). Regarding BPD, therapeutic approaches such as schema therapy (110) may address unmet emotional needs helping to cope with loneliness distress. Besides, analytic therapies, e.g., transference-focused psychotherapy [TFP, (111)] may analyze transference and countertransference processes to identify and integrate primary experiences in dyadic relationships to address loneliness.

Though our findings are valuable for generating a hypothetical model, we are aware that the study has clear limitations: first, due to limited sample sizes, particularly negative findings carry a large beta error. Despite FDR correction, we calculated a large number of correlation analyses. A lower variance in the HC sample (e.g., less CM), may explain the observed diverging results for correlation coefficients and mediation analyses that underline the need to replicate our results in larger samples. For instance, mediation analyses were not performed for each patient group separately due to the small sample size. Similarly, larger sample sizes are needed to clarify whether RS is a general factor in the experience of loneliness or a rather characteristic feature in PDD and BPD patients. Second, as depression is a prevalent comorbidity in BPD patients, findings in both patient groups may rather be related to their depressive symptoms than represent specific characteristics in BPD. Thus, future studies need to disentangle this issue by comparing BPD patients with and without co-morbid depression. Third, as cross-sectional data were used to model longitudinal processes, we cannot draw any conclusions regarding causality. Further, cross-sectional analyses can produce biased estimates of longitudinal processes (112, 113) underlining the need to replicate our findings in a longitudinal design. Fourth, intervention studies could help to dismantle the direction of effect (i.e., do patients report lower levels of loneliness after psychotherapy in which cognitive-affective biases associated with loneliness and possible CM are targeted). In addition, our matched HC groups differed regarding loneliness, as HC_PDD_ reported higher loneliness levels than HC_BPD_. This may explain different correlation patterns, however, HC groups did not differ in this respect for most measures. Finally, our data rely on self-reports, and the reliability of retrospective reports on CM could be questioned. Though subjective recall is an acceptable method as it more likely results in under-reporting of CM than over-reporting (114), a recent meta-analysis suggests that retrospectively self-reported CM might reflect a negative bias (115). In sum, our results should therefore be considered preliminary and interpreted with caution.

## Conclusion

Feelings of loneliness are highly prevalent in PDD and BPD patients and contribute to symptom burden. Therefore, clinicians should pay attention to feelings of loneliness when treating patients with PDD or BPD. Of note, both objective and subjective measures of social isolation should be considered in a complementary way, as they are likely to have an independent effect on mental health. Our findings suggest that clinicians should assess the history of early interpersonal trauma and be aware of the possible presence of high RS when treating PDD or BPD patients. Psychotherapeutic approaches that focus on dysfunctional interpersonal processes and maladaptive social cognitions may be promising in reducing feelings of loneliness. Finally, future studies are needed to validate the hypothetical model of loneliness as proposed here.

## Data Availability Statement

The raw data supporting the conclusions of this article will be made available by the authors, without undue reservation.

## Ethics Statement

The studies involving human participants were reviewed and approved by Ethics committee Faculty of Medicine Ludwig Maximilian University Munich, Munich, Germany EK-No. 281-11. The patients/participants provided their written informed consent to participate in this study.

## Author Contributions

TN-M, BB, JD-K, FP, and MR designed research. TN-M, BB, SG, SR, KZ, RM, AJ, FP, and MR analyzed and interpreted data. TN-M, BB, and MR wrote first draft of manuscript. All authors revised the work critically, approved the final manuscript, and agree to be accountable for the content of the work.

## Conflict of Interest

The authors declare that the research was conducted in the absence of any commercial or financial relationships that could be construed as a potential conflict of interest. The handling editor declared a past co-authorship with several of the authors AJ and FP.
